# Comparison of Anticoagulation Quality between Acenocoumarol and Warfarin in Patients with Mechanical Prosthetic Heart Valves: Insights from the Nationwide PLECTRUM Study

**DOI:** 10.3390/molecules26051425

**Published:** 2021-03-06

**Authors:** Danilo Menichelli, Daniela Poli, Emilia Antonucci, Vittoria Cammisotto, Sophie Testa, Pasquale Pignatelli, Gualtiero Palareti, Daniele Pastori, the Italian Federation of Anticoagulation Clinics (FCSA)

**Affiliations:** 1 I Clinica Medica, Atherothrombosis Centre, Department of Clinical, Internal, Anesthesiological and Cardiovascular Sciences, Sapienza University of Rome, 00185 Rome, Italy; danilo.menichelli@uniroma1.it (D.M.); pasquale.pignatelli@uniroma1.it (P.P.); 2 Thrombosis Centre, Azienda Ospedaliero-Universitaria Careggi, 50134 Florence, Italy; polida@aou-careggi.toscana.it; 3 Arianna Anticoagulazione Foundation, 40138 Bologna, Italy; e.antonucci@fondazionearianna.org (E.A.); gualtiero.palareti@unibo.it (G.P.); 4 Department of General Surgery and Surgical Specialties “Paride Stefanini”, Sapienza, University of Rome, 00185 Rome, Italy; vittoria.cammisotto@uniroma1.it; 5 Department of Laboratory Medicine, Haemostasis and Thrombosis Center, AO Istituti Ospitalieri, 26100 Cremona, Italy; s.testa@ospedale.cremona.it

**Keywords:** warfarin, acenocoumarol, mechanical valve, time in therapeutic range, anticoagulation

## Abstract

Vitamin K antagonists are indicated for the thromboprophylaxis in patients with mechanical prosthetic heart valves (MPHV). However, it is unclear whether some differences between acenocoumarol and warfarin in terms of anticoagulation quality do exist. We included 2111 MPHV patients included in the nationwide PLECTRUM registry. We evaluated anticoagulation quality by the time in therapeutic range (TiTR). Factors associated with acenocoumarol use and with low TiTR were investigated by multivariable logistic regression analysis. Mean age was 56.8 ± 12.3 years; 44.6% of patients were women and 395 patients were on acenocoumarol. A multivariable logistic regression analysis showed that patients on acenocoumarol had more comorbidities (i.e., ≥3, odds ratio (OR) 1.443, 95% confidence interval (CI) 1.081–1.927, *p* = 0.013). The mean TiTR was lower in the acenocoumarol than in the warfarin group (56.1 ± 19.2% vs. 61.6 ± 19.4%, *p* < 0.001). A higher prevalence of TiTR (<60%, <65%, or <70%) was found in acenocoumarol users than in warfarin ones (*p* < 0.001 for all comparisons). Acenocoumarol use was associated with low TiTR regardless of the cutoff used at multivariable analysis. A lower TiTR on acenocoumarol was found in all subgroups of patients analyzed according to sex, hypertension, diabetes, age, valve site, atrial fibrillation, and INR range. In conclusion, anticoagulation quality was consistently lower in MPHV patients on acenocoumarol compared to those on warfarin.

## 1. Introduction

The burden of valvular heart disease (VHD) is still rising worldwide due to degenerative valve diseases. Although valve rheumatic disease is decreasing [1]. Implantation of mechanical prosthetic heart valves (MPHV) is associated with a reduction in valve-related morbidity compared to biological valves [2]. In MPHV, long-term antithrombotic treatment with only vitamin K antagonists (VKAs) is needed [3]. Consolidated evidence from studies including patients with atrial fibrillation (AF) showed that during VKA treatment, a poor anticoagulation quality, expressed as low time in therapeutic range (TiTR) (<65%–70%), was associated with an increased risk of thromboembolism [4], cardiovascular events [5], and mortality [6].

However, no specific indication regarding the type of VKAs, such as warfarin or acenocoumarol, is given by international guidelines or expert consensus documents. As a consequence, the use of different VKAs is highly variable among countries, with warfarin being more commonly used in the United Kingdom and Italy, acenocoumarol in Spain, phenprocoumon in Germany, and fluindione in France [7].

Few previous studies investigated potential differences in patients treated with different VKAs. A study including 498 patients with various indications of anticoagulant therapy (AF in 70% of cases) showed that acenocoumarol may be associated with a lower TiTR and a higher international normalized ratio (INR) variability, which improved after switching to phenprocoumon [8].

Previous evidence showed a generally low quality of anticoagulation with VKAs in patients implanted with MPHV [9], but the difference between warfarin and acenocoumarol in terms of clinical characteristics of patients and anticoagulation quality was not investigated in these patients.

The aims of our study were (1) to investigate the clinical characteristics of patients treated with acenocoumarol compared to those treated with warfarin, (2) to describe clinical determinants associated with acenocoumarol use, and (3) to report the proportion of suboptimal anticoagulation quality in acenocoumarol and warfarin use in patients enrolled in the multicenter PLECTRUM registry.

## 2. Results

The study enrolled 2111 patients with MPHV, of which 1716 (81.3%) were treated with warfarin and 395 (18.7%) with acenocoumarol. The mean age was 56.8 years and 44.6% of patients were women (Table 1).

The MPHV site most represented in the whole cohort was aortic (60.7%) and 38.4% of patients had concomitant AF. Patients on acenocoumarol were more frequently affected by arterial hypertension, heart failure (HF), and peripheral artery disease (PAD) and had more comorbidities compared to those on warfarin (Table 1). There was no difference between anticoagulant treatment groups concerning age, sex, MPHV site, INR range, diabetes, previous ischemic heart disease, or thromboembolism at baseline (Table 1). Patients treated with acenocoumarol were affected by a higher number of comorbidities at baseline compared to those treated with warfarin (26.6% vs. 20.0%, respectively, *p* = 0.004).

At univariable logistic regression analysis (Table 2), factors associated with acenocoumarol use were the number of comorbidities, in particular arterial hypertension, PAD, and HF. At multivariable logistic regression analysis, only the presence of three or more comorbidities (OR 1.443, 95%CI 1.081–1.927, *p* = 0.013) were associated with acenocoumarol use. In a second model using single comorbidities, we found that PAD (OR 1.536, 95%CI 1.085–2.174, *p* = 0.015) and arterial hypertension (OR 1.292, 95%CI 1.016–1.642, *p* = 0.036) were associated with acenocoumarol use.

### Anticoagulation Quality According to Treatment

In the whole cohort, the mean TiTR was 60.6 ± 19.5%; anticoagulation quality was lower in patients treated with acenocoumarol compared to those on warfarin (61.6 ± 19.4% vs. 56.1 ± 19.2%, *p* < 0.001, Table 1).

Analyzing the proportion of suboptimal anticoagulation using different cutoffs of TiTR, we found that acenocoumarol users had a higher prevalence of TiTR < 60%, <65%, or <70% (*p* < 0.001 for all comparisons, Table 1).

**Figure 1:**
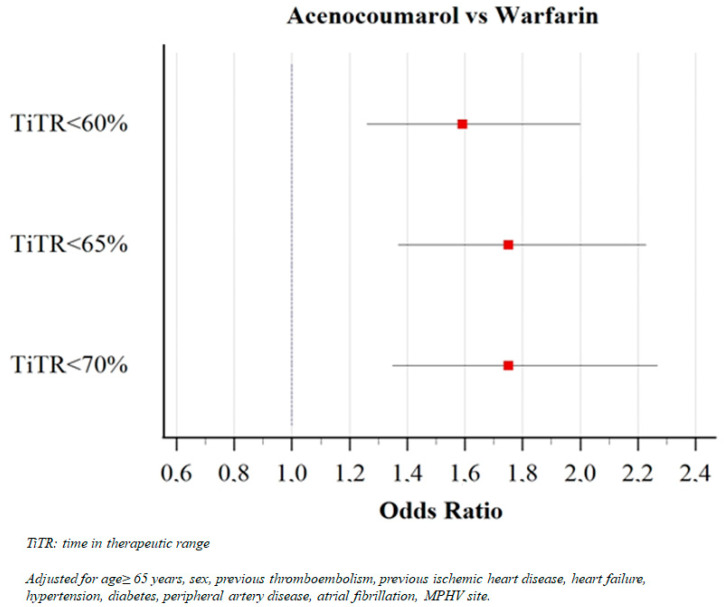
Association among acenocoumarol and low time in therapeutic range using different cutoff values in multivariable regression analysis.

Furthermore, after performing a multivariable regression analysis of factors associated with poor anticoagulation quality, acenocoumarol use was found to be associated with low TiTR regardless of the cutoff used: TTR < 60% (OR 1.590, 95%CI 1.262–2.002, *p* < 0.001), TTR < 65% (OR 1.747, 95%CI 1.368–2.232. *p* < 0.001), and TTR < 70% (OR 1.747, 95%CI 1.347–2.266, *p* < 0.001) (Figure 1).

To better characterize the association between acenocoumarol and low TiTR, we performed a subgroup analysis according to sex, hypertension, diabetes, age (≥65 years), MPHV site, AF, and INR range (Table 3). A lower anticoagulation quality on acenocoumarol was found in all subgroups of patients analyzed (Table 3).

We repeated the analysis, excluding patients treated with antiplatelets, and found similar results in 1746 patients as follows: 56.2 ± 18.8 in acenocoumarol-treated vs. 61.7 ± 19.2 in warfarin-treated patients (*p* < 0.001).

## 3. Material and Methods

The FCSA-START Valve Study (PLECTRUM) is a retrospective multicenter observational study conducted within the Italian Survey on Anticoagulation Patient Records (START register) and conducted among 33 centers affiliated with the Italian Federation of Thrombosis Diagnosis Centers and Surveillance of Antithrombotic Therapies (FCSA) [10]. The centers were asked to select from their databases patients with a mechanical heart valve prosthesis that was implanted after 1990 and who were followed up on for the management of oral anticoagulant therapy. Patients with MPHV were treated with warfarin or acenocoumarol to prevent thromboembolic event according to European Society of Cardiology guidelines [11]. Each physician prescribed warfarin or acenocoumarol after individualized clinical evaluation. The patients followed by the FCSA centers for the management of oral anticoagulation received an adequate education on the purpose of the treatment, the risk of complications, the INR values, and the management of the dosage of the drugs. The centers performed periodic INR measurements based on INR value, prescribe daily VKA, dosage and scheduled the date for subsequent visits; they also monitored and recorded changes in patient habits, diet, co-medications, intercurrent illness, bleeding, and thrombotic complications during regular follow-up visits through patient interviews. All centers participated in the specially designed external laboratory quality control program, which is performed 3 times a year and uses lyophilized plasma samples obtained from anticoagulated patients. For this reason, to standardize the quality of INR measurements, none of the patients were monitored at home.

Demographic information and clinical data were collected. Patients were classified as having high blood pressure if they were taking medicines to lower their blood pressure. Diabetes mellitus was defined according to the criteria of the American Diabetes Association. Coronary artery disease was defined on the basis of a history of myocardial infarction or stable and unstable angina. Heart failure was defined as the presence of signs and symptoms of right or left ventricular failure or both and confirmed by non-invasive or invasive measurements that demonstrated objective evidence of cardiac dysfunction. The quality of the anticoagulant control, calculated as TiTR using the linear interpolation method of Rosendaal et al. [12], was analyzed considering the INRs recorded in the last year of follow-up. The study protocol complied with the ethical guidelines of the 1975 Helsinki Declaration, as evidenced by the approval of the institution’s human research committee, and informed consent was obtained from each patient. Authorization to set up the registry was obtained from the Ethical Committee of the University Hospital “S. Orsola-Malpighi,” Bologna, Italy, in October 2011 (N = 142/2010/0/0ss”). The same institution is charged with deploying and upkeeping the registry central database.

### Statistical Analysis

Continuous variables were reported as mean and standard deviation and compared by the Student t-test. Categorical variables were reported as count and percentage and compared by Pearson chi-squared test. A first descriptive analysis of clinical characteristics according to acenocoumarol or warfarin use was performed. Univariable and multivariable logistic regression analysis was used to calculate the relative odds ratio (OR) with a 95% confidence interval (95%CI) of factors associated with acenocoumarol use and low TiTR. Significance was set at a *p*-value < 0.05. All tests were two-tailed and analyses were performed using computer software packages (SPSS-25.0, SPSS Inc. IBM Corp, Armonk, NY, USA).

## 4. Discussion

The difference between acenocoumarol and warfarin effectiveness in terms of anticoagulation stability was never investigated in a large cohort of patients with MPHV. Findings from our study show that 18.7% of patients implanted with MPHV were treated with acenocoumarol in specialized outpatients’ clinics for the management of antithrombotic therapies. Acenocoumarol prescription was more common in complex patients, as indicated by the higher number of comorbidities. Patients treated with acenocoumarol showed lower anticoagulation quality compared to those on warfarin. This difference was consistent in all thresholds of TiTR used and in all subgroups of patients regardless of sex, age, valve site, or INR range.

Acenocoumarol presents some pharmacokinetic and pharmacodynamic differences from warfarin that may turn useful in some patients, such a more rapid onset of action, a shorter half-life, and lower renal excretion. In our study, patients with a higher number of comorbidities and use of antiplatelet agents were more frequently prescribed acenocoumarol instead of warfarin. In this last context, the shorter half-life of acenocoumarol may be an advantage in the case of a major or life-threatening bleeding event in patients treated with dual therapy needing a rapid offset of action of the drug.

We found a generally lower anticoagulation quality in patients treated with acenocoumarol, which persisted after adjustment for confounders. Suboptimal anticoagulation with acenocoumarol compared to warfarin was also consistent in all subgroups of patients analyzed, such as sex, hypertension, diabetes (mostly for TiTR < 60%), AF, MPHV site, and INR range. This finding adds to previous evidence that female sex is associated with lower overall anticoagulation quality in the PLECTRUM registry [13].

In a study performed in Poland including 430 patients with mixed indications for VKAs therapy (65.8% AF, 22.6% venous thromboembolism, and 11.6% MPHV) and treated in most cases with acenocoumarol (78.8%), the mean TiTR was as low as 55%, with acenocoumarol use associated with a nearly threefold higher chance of having INR outside the therapeutic range [14].

A previous small study including patients with various indications of anticoagulation showed a significant improvement of TiTR in patients switched from acenocoumarol to warfarin (from 40.2% to 60.2%) [15].

Furthermore, in a population with similar age affected by venous thromboembolism enrolled within the EINSTEIN-DVT and EINSTEIN-PE studies, the use of acenocoumarol was a risk factor for long-term low TiTR (OR 1.81, 95%CI 1.49–2.20, *p* < 0.01) [16].

As patients treated with acenocoumarol were more frequently prescribed antiplatelet agents, which may lead to an increased risk of bleeding episodes and subsequently to a lower adherence to anticoagulant prescription and to a higher discontinuation rate [17], we also performed a subgroup analysis excluding patients on antiplatelets. In this group of patients, we found similar results than the overall cohort, suggesting that anticoagulation quality in MPHV patients is not affected by concomitant administration of antiplatelet drugs.

Our results may have implications for clinical practice. Prescribing acenocoumarol or switching from warfarin should be considered only in select patients in whom warfarin therapy is not successful, such as those with low TiTR or those with recurrent thrombotic events; in patients with a known or suspected warfarin resistance [18], such as antiphospholipid syndrome [19]; and in patients taking drugs interacting with warfarin metabolism.

Our study has limitations to be acknowledged. First, its retrospective observational design is an intrinsic limitation to establishing any inference on our observation. Second, some additional factors not considered in this study may affect both the choice of acenocoumarol use and TiTR; for instance, use of different VKAs may be affected by national guidelines in different countries. Furthermore, some drugs interacting with VKAs that were not considered in this study may influence the TiTR. Finally, we do not have data on concomitant hospitalizations and interruptions for diagnostic/therapeutic procedures that may lead to low anticoagulation quality. In addition, we included only Caucasian patients with a mean age of 60 years, and the reproducibility of our findings in elderly patients and in patients with different ethnic origins needs to be explored. Indeed, ethnic differences such as environmental factors and genetic variants of isoenzymes may affect pharmacokinetic features, hepatic metabolism, and renal elimination of warfarin [20]. Finally, the difference between acenocoumarol and warfarin in other settings such as AF and venous thrombosis needs to be explored, even if in these contexts the use of direct oral anticoagulation is replacing VKAs in many countries.

In conclusion, warfarin would be the first-choice treatment for thromboprophylaxis in patients with MPHV regardless of valve site and INR range. Switching from acenocoumarol to warfarin may improve TiTR in patients with unstable anticoagulation.

## Figures and Tables

**Table 1:** Characteristics of patients according to vitamin K antagonists.

	Whole Cohort (n = 2111)	Warfarin (n = 1716)	Acenocoumarol (n = 395)	*p*-Value
**Age (years)**	56.8 ± 12.3	56.8 ± 12.3	56.9 ± 12.2	0.869
**Age ≥ 65 years (%)**	29.1	28.6	31.1	0.319
**Age ≥ 75 years (%)**	4.0	4.0	4.1	0.978
**Women (%)**	44.6	44.4	45.3	0.743
**Arterial hypertension (%)**	65.9	64.7	70.9	0.020
**Diabetes (%)**	13.5	13.2	14.7	0.445
**Heart failure (%)**	14.9	14.2	18.2	0.041
Previous thromboembolism * (%)	7.8	7.5	9.4	0.203
**Previous hemorrhage**	3.8	4.2	2.3	0.074
**Previous ischemic heart disease (%)**	12.9	12.6	14.2	0.413
**Previous clinical outcomes ^**	5.8	6.1	4.3	0.163
Peripheral artery disease ** (%)	9.0	8.2	12.4	0.008
**Atrial fibrillation (%)**	38.4	38.3	39.0	0.796
**Comorbidities ^§^**	1.4 ± 1.0	1.4 ± 1.0	1.6 ± 1.1	0.004
**Comorbidities ≥ 3**	14.5	13.6	18.5	0.013
**Concomitant antiplatelet (%)**	17.3	16.6	20.5	0.061
**Amiodarone users (%)**	13.2	13.3	12.4	0.618
**MPHV site**				0.379
**Aortic (%)**	60.7	60.6	61.0	
**Mitral (%)**	28.1	28.6	26.1	
**Mitroaortic (%)**	11.2	10.8	12.9	
**INR ranges**				0.898
**2.0–3.0 (%)**	27.2	27.4	26.3	
**2.5–3.5 (%)**	63.6	63.4	64.5	
**3.0–4.0 (%)**	9.2	9.2	9.2	
**TiTR (%)**	60.6 ± 19.5	61.6 ± 19.4	56.1 ± 19.2	<0.001
**Low-quality anticoagulation**				
**TiTR < 60% (%)**	48.5	46.3	58.0	<0.001
**TiTR < 65% (%)**	60.1	57.8	70.4	<0.001
**TiTR < 70% (%)**	66.9	64.8	76.2	<0.001

INR: international normalized ratio. MPHV: mechanical prosthetic heart valve. TiTR: time in therapeutic range. * Includes previous stroke/TIA/systemic embolism. ** Includes lower limb and carotid disease. ^ Previous thromboembolism, previous ischemic heart disease, previous hemorrhage. ^§^ Includes hypertension, diabetes, heart failure, peripheral artery disease, atrial fibrillation.

**Table 2:** Univariable logistic regression analysis of clinical factors associated with acenocoumarol use.

	Odds Ratio	95% Confidence Interval	*p*-Value
**Female sex**	0.964	0.774–1.201	0.743
**Age ≥ 65 years**	0.886	0.669–1.124	0.319
**Atrial fibrillation**	1.030	0.823–1.289	0.796
**Hypertension**	1.326	1.044–1.683	0.021
**Diabetes**	1.129	0.827–1.542	0.446
**PAD**	1.594	1.128–2.252	0.008
**Heart failure**	1.351	1.012–1.804	0.041
**Previous TE**	1.282	0.874–1.881	0.204
**Previous ischemic heart disease**	1.141	0.831–1.566	0.414
**Previous hemorrhage**	0.532	0.264–1.074	0.078
**Comorbidities ≥ 3**	1.443	1.081–1.927	0.013
**Previous clinical outcomes**	0.690	0.408–1.166	0.166
**Mitral vs. Aortic**	0.907	0.703–1.170	0.453
**Mitroaortic vs. Aortic**	1.183	0.842–1.662	0.332
**Concomitant antiplatelet**	1.301	0.988–1.713	0.061
**Amiodarone**	0.920	0.661–1.279	0.619

PAD: peripheral artery disease. TE: Thromboembolism.

**Table 3:** Subgroup analysis of time in therapeutic range according to acenocoumarol or warfarin use.

	OAC Type	Mean TiTR	*p*	TiTR < 60% (%)	*p*	TiTR < 65% (%)	*p*	TiTR < 70% (%)	*p*
**Women**	*Warfarin*	58.9 ± 19.1	<0.001	52.4	0.001	63.4	<0.001	70.6	0.003
	*Acenocoumarol*	51.9 ± 18.6		65.9		77.7		81.6	
**Men**	*Warfarin*	63.8 ± 19.4	0.004	41.5	0.008	53.2	0.003	60.2	0.002
	*Acenocoumarol*	59.6 ± 19.0		51.4		64.4		71.8	
**Arterial hypertension**	*Warfarin*	60.6 ± 19.8	<0.001	49.7	<0.001	60.6	<0.001	67.2	<0.001
	*Acenocoumarol*	54.8 ± 19.1		62.9		73.9		79.3	
**Diabetes**	*Warfarin*	57.8 ± 19.3	0.019	56.8	0.016	66.1	0.052	70.5	0.060
	*Acenocoumarol*	51.1 ± 19.9		74.1		79.3		82.8	
**Age (** **≥** **65 years)**	*Warfarin*	60.2 ± 18.9	0.001	48.9	0.006	61.3	0.001	68.2	0.004
	*Acenocoumarol*	53.6 ± 18.4		62.6		77.2		81.3	
**Aortic MPHV**	*Warfarin*	65.0 ± 19.3	<0.001	37.8	<0.001	49.6	<0.001	57.2	<0.001
	*Acenocoumarol*	59.5 ± 18.9		50.2		63.9		69.7	
**Mitral/Mitroaortic MPHV**	*Warfarin*	56.4 ± 18.5	0.001	59.5	0.014	70.3	0.010	76.5	0.007
	*Acenocoumarol*	50.8 ± 18.4		70.1		80.5		86.4	
**INR range 2.0–3.0**	*Warfarin*	70.3 ± 19,0	0.026	26.5	0.125	37.4	0.014	43.6	0.012
	*Acenocoumarol*	65.7 ± 19.1		34.0		50.5		57.3	
**INR range above 2.0–3.0**	*Warfarin*	58.4 ± 18.6	<0.001	53.8	<0.001	65.4	<0.001	72.8	<0.001
	*Acenocoumarol*	52.7 ± 18.1		66.4		77.4		82.9	
**Atrial fibrillation**	*Warfarin*	58.5 ± 19.4	0.002	41.9	0.002	53.4	<0.001	61.1	0.001
	*Acenocoumarol*	53.1 ± 19.0		53.1		67.2		72.6	

MPHV: mechanical prosthetic heart valve; OAC: oral anticoagulant; TiTR: time in therapeutic range.

## Data Availability

The data presented in this study are available in this article.

## Sample Availability

Not available.

## Appendix A

Italian Federation of Anticoagulation Clinics (FCSA).

Sophie Testa, Oriana Paoletti; Dipartimento di Medicina di Laboratorio, Centro Emostasi e Trombosi ASST Cremona.

Corrado Lodigiani; Paola Ferrazzi; Ilaria Quaglia; Centro Trombosi e Malattie Emorragiche, Humanitas Research Hospital, IRCCS Rozzano-Milano.

Daniela Poli Centro Trombosi SOD Malattie Aterotrombotiche Azienda Ospedaliero Universitaria Careggi; Firenze.

Nadia Coffetti; Rosa Marotta; Varusca Brusegan, Orazio Bergamelli, Servizio di Immunoematologia e Medicina Trasfusionale Azienda Ospedaliera Bolognini, ASST Bergamo Est, Seriate e Ambulatorio TAO SIMT Ospedale Fernaroli, Alzano Lombardo.

Roberto Facchinetti Laboratorio Analisi Azienda Ospedaliera Universitaria Integrata Ospedale Civile Maggiore Di Borgo Trento; Verona.

Giuseppina Serricchio; Silvia Sarpau, Francesca Brevi; ASST Lariana Como.

Pietro Falco; Guarino Silverio; Poliambulatorio Specialistico MEDICAL PONTINO, Latina.

Catello Mangione; Giacomo Bellomo, Servizio Immunotrasfusionale Ospedale “Santa Caterina Novella” Galatina (Lecce).

Serena Masottini; Alessandra Cosenza; Centro per la prevenzione, diagnosi e trattamento dellemalattie tromboemboliche- Asl 8- Cagliari.

Lucia Ruocco; Paolo Chiarugi, Monica Casini; Ambulatorio Antitrombosi CAT-TAO AOU Pisana, Pisa.

Arturo Cafolla; Antonietta Ferretti; Ematologia, UOS Emostasi e Trombosi, Policlinico Umberto I°, Roma.

Giorgia Micucci; Serena Rupoli; Lucia Canafoglia; Azienda Ospedaliero-Universitaria Ospedali Riuniti Di Ancona.

Paolo Pedico; Rita Galasso; Rosa Rotunno; U.O. MedicinaTrasfusionale Ospedale Mons. Raffaele Dimiccoli Barletta.

Antonio Insana; Paolo Valesella; Servizio Di Patologia Clinica Dipartimento Dei Servizi—Ospedale S. Croce Moncalieri, Moncalieri, Torino.

Angelo Santoro; U.O.C. Patologia Clinica e Centro Trombosi, Presidio Ospedaliero “A. Perrino,” ASL Brindisi.

Francesco Marongiu, Doris Barcellona; Centro Di Fisiopatologia dell’emostasi e Terapia Anticoagulante, Azienda Ospedaliera Universitaria di Monserrato, Cagliari.

Vittorio Pengo, Gentian Denas; Istituto di Cardiologia, Policlinico Universitario, Università di Padova.

Carmelo Paparo; Centro Anti Trombosi Ospedale Maggiore ASL TO5, Chieri (Torino).

Eugenio Bucherini; Flavia Tani; Enrico Carioli, Centro di Sorveglianza per la Terapia Anticoagulante, Angiolgia- Medicina Vascolare U.O. Cardiologia O.C. Per gli infermi—Faenza AUSL Romagna, (Ravenna).

Francesco Violi, Pasquale Pignatelli, Daniele Pastori, Mirella Saliola; Dipartimento di Scienze Cliniche, Internistiche, Anestesiologiche e Cardiovascolari, Sapienza Università di Roma.

Lucilla Masciocco, Pasquale Saracino, Angelo Benvenuto, UOC Medicina Interna, Centro Controllo Coagulazione, Ospedale Lastaria, Lucera (Foggia).

Anna Turrini; Stefano Ciaffone; Ospedale “SACRO CUORE” Laboratorio Analisi Cliniche E Medicina Trasfusionale Negrar, Verona.

Andrea Toma; Pietro Barbera UOC di Patologia Clinica Arzignano, Vicenza.

Paolo Gresele; Tiziana Fierro; Stefano Pasquino Department of Medicine, Section of Internal and Cardiovascular Medicine, University of Perugia.

Lucia La Rosa; Rino Morales Centro Trasfusionale e ambulatorio emostasi e trombosi; ASST Vimercate.

Francesco Ronchi, Giuseppe Isu Centro Tao Servizio Di Patologia Clinica Ospedale Ns Signora Di Bonaria Asl 6 Sanluri.

Teresa Lerede, Luca Barcella Centro Trombosi e Emostasi Immunoematologia eMedicina Trasfusionale ASST Papa Giovanni XXIII, Bergamo.

Luigi Ria, Centro Trombosi ed. Emostasi, U.O.C. di Medicina Interna, Ospedale “Sacro Cuore di Gesù” Gallipoli; ASL Lecce, Lecce.

Rosanna Crisantemo; Luciano Suriano, Luciano Lorusso; Mario De Sarlo Servizio di Immunoematologia e Medicina Trasfusionale Ospedale L.Bonomo, Andria.

Pasquale Carrato Istituto Polidiagnostico Santa Chiara, Agropoli, Salerno.

Carmine Oricchio U.O.S. Centro Trasfusionale del P.O. “Luigi Curto” di Polla—ASL Salerno.

Elvira Grandone, Donatella Colaizzo, Centro Trombosi, I.R.C.C.S. Casa Sollievo della Sofferenza, S. Giovanni Rotondo, Foggia.

Maurizio Molinatti Unità Funzionale di Ematologia Centro TAO Humanitas Cell, Torino.
